# Risk factors for iliac limb migration after endovascular infrarenal aortic repair

**DOI:** 10.1038/s41598-025-92488-6

**Published:** 2025-03-04

**Authors:** Byung Chan Lee, Chan Park, Hyoung Ook Kim, Woong Yoon, Yong Yeon Jeong, Soo Jin Na Choi, Ho Kyun Lee, Hong Sung Jung, Youngsup Yoo

**Affiliations:** 1 Department of Radiology, Chonnam National University Hwasun Hospital, 322 Seoyang-ro, Hwasun, 58128 Korea; 2 Department of Radiology, Chonnam National University Hospital, 42 Jebong-ro, Dong-gu, Gwangju, 61469 Korea; 3 Department of Surgery, Chonnam National University Hospital, 42 Jebong-ro, Dong-gu, Gwangju, 61469 Korea; 4 Department of Radiology, Chonnam National University Medical School, 160 Baekseo-ro, Dong-gu, Gwangju, 61469 Korea; 5 Department of Surgery, Chonnam National University Medical School, 160 Baekseo-ro, Dong-gu, Gwangju, 61469 Korea

**Keywords:** Iliac limb migration, Endovascular aneurysm repair (EVAR), Type 1b endoleak, Common iliac artery diameter, Iliac limb oversizing, Postoperative surveillance, Aneurysm, Aortic diseases

## Abstract

This study investigated anatomical and procedural factors influencing iliac limb migration and its correlation with late type 1b and type 3 endoleaks. We analyzed data of 141 iliac limbs from 91 patients who underwent endovascular aneurysm repair for infrarenal abdominal aortic aneurysm between 2005 and 2017. Iliac limb migration was measured using initial and follow-up computed tomography angiography scans conducted at least three years post-procedure, with significant migration defined as a change of ≥ 5 mm. The iliac limbs were classified into Group 1 (G1; *n* = 34 limbs, 26 patients) with significant migration and Group 2 (G2; *n* = 107 limbs, 65 patients) without significant migration. The median follow-up periods were 70.5 months (interquartile range 49.7–91.8 months) for G1 and 57.6 months (interquartile range 44.2–73.2 months) for G2. Multivariable analysis confirmed that significant migration correlated with larger common iliac artery (CIA) diameters and lower iliac limb oversizing. Significant iliac limb migration was associated with a higher risk of type 1b endoleak development. Our findings suggest that careful iliac limb oversizing is essential for patients with a CIA diameter > 20 mm, and vigilant monitoring of the iliac landing zone is crucial during postoperative surveillance.

## Introduction

Endovascular aneurysm repair (EVAR) has emerged as the primary treatment for abdominal aortic aneurysm (AAA) in patients with suitable anatomical characteristics^1^. However, concerns remain regarding the long-term durability of EVAR, particularly due to the higher frequencies of reinterventions and delayed complications^2–5^.

One significant drawback of EVAR is stent graft migration, which can cause severe complications such as type 1 endoleak, stent graft separation, kinking, or graft occlusion. This issue is a primary factor contributing to the need for EVAR reinterventions^6–8^. While much research has focused on proximal stent graft migration at the aortic neck^9–12^, there is less attention on the cephalad migration of iliac limb stent grafts at the distal landing zone.

Several studies have suggested that the migration of iliac stent grafts can be influenced by factors such as large AAAs, dilated or aneurysmal common iliac artery (CIA), short lengths of CIA fixation, lower degrees of iliac limb oversizing, and bell-bottom iliac limbs^8,13–16^. However, studies specifically assessing risk factors contributing to significant iliac limb migration at the distal landing zone remain scarce, with underexplored complex interactions among these factors. Consequently, this study investigates the anatomical and procedural factors associated with iliac limb migration and examines the potential correlation between iliac limb migration and the occurrence of type 1b and type 3 endoleaks.

## Methods

### Patients

This retrospective study, conducted at a single center (Chonnam National University Hospital, Gwangju, Korea), was approved by the Institutional Review Board (IRB) of Chonnam National University Hospital. Due to the retrospective nature of the study, the IRB (CNUH-2024-103) waived the requirement of obtaining informed consent. All methods were performed in accordance with the relevant guidelines and regulations. The study included all patients who underwent EVAR for infrarenal AAA between 2005 and 2017. The following patients were excluded from the study: First, patients who did not undergo preoperative computed tomography angiography (CTA) within three months before EVAR were excluded to ensure accurate and up-to-date anatomical assessment. Second, to ensure accuracy in evaluating procedural factors and to establish a reliable baseline for assessing iliac limb migration, patients who did not undergo postoperative CTA within one month of the procedure were excluded. Third, long-term imaging data were necessary to analyze iliac limb migration and assess outcomes over time. Consequently, patients who did not undergo postoperative CTA at least three years after EVAR were excluded to ensure the reliability of migration-related findings. Fourth, patients treated with straight aortic endografts were excluded from the study because their design does not involve iliac limb placement, which is the focus of this study. Fifth, aorto-uni-iliac (AUI) systems extend into a single iliac artery via a tapered tube and are usually paired with a contralateral iliac occluder and femorofemoral crossover graft. They are typically indicated in cases where adverse iliac anatomy is present, such as tortuous, stenotic, or aneurysmal vessels. Furthermore, these devices may be necessary when a calcified and stenotic aortic bifurcation obstructs the proper deployment of the two iliac limbs of a bifurcated device. To minimize bias resulting from these procedural differences and anatomical variations and to achieve a more homogeneous sample for analysis, patients treated with AUI endografts were excluded. Sixth, patients lacking detailed stent-graft sizing data were excluded, as such information is critical for assessing procedural factors, including iliac limb oversizing. Seventh, patients with early type 1 or type 3 endoleaks occurring within six months were excluded, as these complications arise from anatomical or technical factors unrelated to iliac limb migration and typically require immediate reintervention. However, type 1 or 3 endoleaks detected beyond six months post-EVAR were not excluded. Eighth, iliac limbs that became occluded during follow-up were excluded from the analysis because accurately measuring the migration distance of iliac limbs using the Aquarius iNtuiton workstation (TeraRecon, Durham, NC, USA) becomes challenging in these cases. Furthermore, these cases often require immediate reintervention or surgical intervention. Ultimately, all iliac limbs that were extended to the external iliac arteries (EIAs) during EVAR were excluded from the analysis, including those that underwent hypogastric artery embolization or were treated with iliac branch devices. However, if a patient had one iliac limb extended to the EIA while the contralateral limb was deployed within the CIA, only the limb with a landing zone in the CIA was included in the analysis. This criterion was applied to maintain methodological uniformity and enhance sample homogeneity.

### Study protocol

#### Iliac limb migration assessment

**Fig. 1:**
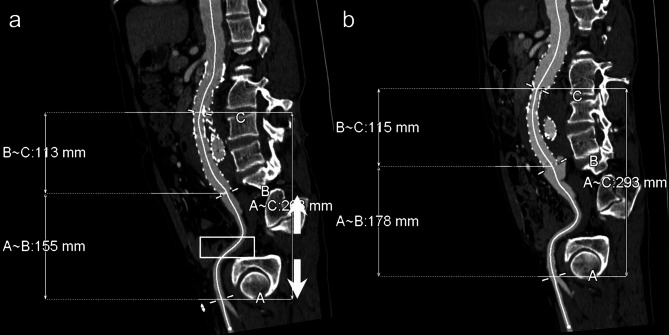
Assessment of limb migration length in a 63-year-old male patient with an infrarenal abdominal aortic aneurysm following endovascular aortic repair. (**a**) The initial post-operative CTA scan, performed one week after the procedure, measured the distance from the origin of the deep femoral artery to the distal end of the left iliac limb stent-graft as 155 mm. (**b**) At the 3-year and 8-month follow-up, the distance was measured at 178 mm on the CTA. The limb migration length was determined to be 23 mm. *CTA* computed tomography angiography.

Iliac limb migration was assessed by two independent researchers who were not involved in the treatment or follow-up processes and were unaware of patient identities. The assessment was conducted using the Aquarius iNtuiton workstation (TeraRecon, Durham, NC, USA). To ensure consistent and accurate measurements, a central lumen-line reconstruction methodology was employed. Central lumen lines were initially reconstructed automatically on the dedicated workstation; any detected errors were corrected manually using a semi-automatic approach. The researchers measured the distance from the origin of the deep femoral artery to the distal end of the iliac limb stent-graft on the initial post-operative CTA within one month and on the final follow-up CTA at a minimum of three years post-EVAR. Significant migration of the iliac limb was defined as a length change of 5 mm or more (Fig. 1). For iliac limbs without significant migration, migration measurements were limited to the initial post-operative CTA and the last follow-up CTA. However, for iliac limbs exhibiting significant migration, additional migration measurements were performed on all interim CTA scans obtained between the initial post-operative and final follow-up CTAs to determine when significant migration was first detected.

#### Anatomical and procedural evaluations

Anatomical characteristics were assessed by two independent investigators who were not involved in the iliac limb migration assessment. Each investigator analyzed a distinct subset of patients. Prior to data collection, a calibration process was conducted to standardize measurement techniques and ensure consistency. Reliability was assessed through periodic cross-checks on 20% of cases, where both investigators independently measured the same parameters. Any discrepancies or ambiguities identified during these cross-checks were resolved through discussion and consensus. This methodology was designed to enhance measurement reliability, minimizing interobserver variability. Anatomical characteristics included the maximum diameter of the infrarenal aortic aneurysm sac, aortic tortuosity index, infrarenal aortic angle, maximum diameter and length of the CIA, iliac tortuosity index, and iliac angle. Pre-procedural CTA measurements were conducted using the Aquarius iNtuiton workstation. The measurements of the aortic tortuosity index, infrarenal aortic angle (previously referred to as the aortic angle in prior study), iliac tortuosity index, and iliac angle followed the methodology described by Chaikof et al.^17^. The infrarenal aortic angle was defined as the most acute angle between the lowest renal artery and the aortic bifurcation, as described in the Anatomic Severity Grading (ASG) system^17^. Procedural factors were also assessed by the same investigators and included the length of the iliac seal, the degree of the iliac seal, the degree of iliac limb oversizing, the diameter of the iliac limb, whether the iliac limbs were deployed in a Ballerina configuration, the manufacturers of the iliac limb, and the occurrence of type 2 endoleak during follow-up. The length of the iliac seal was assessed using established methods^18^ on the initial post-operative CTA. To ensure a precise evaluation of sealing effectiveness, the iliac seal length was defined as the segment of the CIA where the iliac limb exhibited good circumferential apposition with the arterial wall, as described in a prior study^18^. The degree of the iliac seal was quantified by calculating the relative difference between the length of the iliac seal measured on the initial follow-up CTA (reflecting good circumferential apposition of the iliac limb) and the total length of the CIA measured on the pre-procedural CTA, using the formula: (iliac seal length – native CIA length) / native CIA length. Similarly, the degree of iliac limb oversizing was quantified by determining the percentage difference between the diameter of the selected iliac limb and the diameter of the native CIA, using the formula: (iliac limb diameter – native CIA diameter) / native CIA diameter.

Each iliac limb was evaluated separately and categorized into two groups: Group 1 (G1) consisted of iliac limbs with significant migration (≥ 5 mm), while Group 2 (G2) comprised those without significant migration (< 5 mm).

### Statistical analysis

Statistical power analysis for the final sample size was conducted using G*Power 3.1 software^19^. An effect size of 0.5 was selected, representing a moderate effect according to Cohen’s conventions, to detect a statistically significant difference between groups. The alpha level was set at 0.05 to minimize the risk of a Type I error. Subsequently, the statistical analysis was conducted using SPSS Statistics, Version 27 (IBM, SPSS Inc., Chicago, IL, USA). Data normality was assessed using the Kolmogorov-Smirnov test. Data are presented as mean and standard deviation for normally distributed data, median and interquartile range (25th and 75th percentiles) for non-normally distributed data, or as counts and percentages. Anatomical characteristics and procedural factors of both groups were assessed using the Student t-test, Mann-Whitney U test, chi-square test, and Fisher’s exact test as appropriate. To assess the rate of iliac limb migration over time, the migration rate per year was calculated by dividing the total migration distance (mm) by the follow-up duration (years). Additionally, Kaplan-Meier analysis with the log-rank test was used to estimate the time to the first detection of significant migration. To predict the likelihood of significant iliac limb migration, univariate and multivariate Cox proportional hazard models were employed. Variables for multivariate analysis were selected based on a combination of clinical relevance and statistical significance identified in the univariate analysis. Specifically, variables with a p-value of less than 0.05 in the univariate analysis were included in the multivariate model. To minimize the risk of overfitting, the final model was constructed using a backward stepwise selection approach. Multicollinearity was assessed using variance inflation factors (VIFs); variables with a VIF greater than 10 were excluded to ensure the robustness of the model. A p-value below 0.05 was considered statistically significant. Variables that remained significant in the multivariate analysis were further analyzed using Receiver Operating Characteristic (ROC) curve analysis. Optimal cutoff values were determined by maximizing Youden’s index, and limbs were subsequently divided into two groups based on these cutoff values. Survival curves for these groups were assessed using the Kaplan-Meier method. To compare significant iliac limb migration based on the stent-graft type, a subgroup analysis was performed using Kaplan-Meier analysis and the log-rank test for the two most frequently used stent-graft types among the four included in this study. The occurrence of late type 1 and type 3 endoleaks, which manifested more than six months after the procedure, was compared between G1 and G2 using the chi-square test. The temporal relationship between significant migration and the development of type 1b endoleaks was investigated using Kaplan-Meier survival analysis. This analysis assessed the time interval from significant iliac limb migration to the detection of type 1b endoleaks. Additionally, a correlation analysis was conducted to evaluate the association between the timing of significant migration and the detection of type 1b endoleaks. To further investigate anatomical changes over time, additional analyses were conducted to assess variations in CIA diameter. The Mann-Whitney U test was used to compare pre-procedural CIA diameter, last follow-up CIA diameter, and the change in CIA diameter (last follow-up – pre-procedural) between groups with significant iliac limb migration (G1) and those without (G2). Similarly, comparisons were made between groups with type 1b endoleak (G3) and those without (G4) to examine the association between type 1b endoleak and CIA dilation. The correlation between iliac limb migration length and CIA diameter change was assessed using Spearman’s correlation, while the relationship between CIA diameter change and the occurrence of type 1b endoleak was evaluated using both Kendall’s tau-b and Spearman’s rho.

## Results

### Patient and limb characteristics

**Fig. 2:**
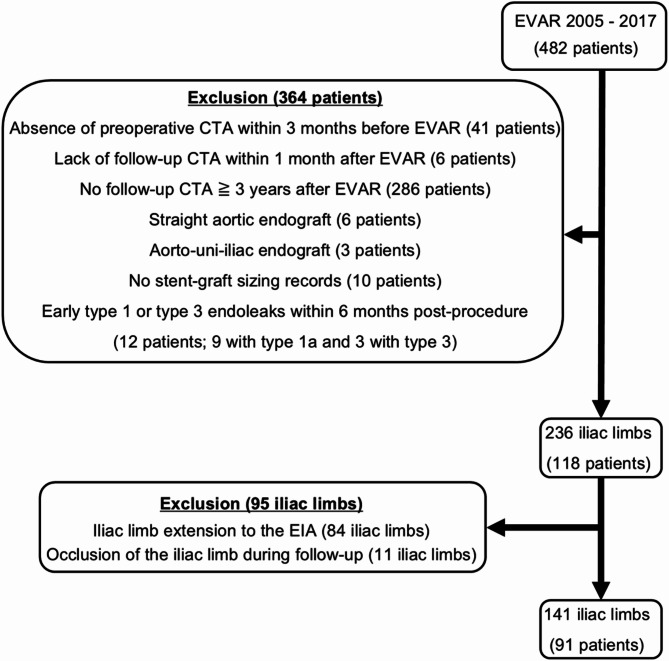
Flow chart for patient and iliac limb selection. *CTA* computed tomography angiography, *EIA* external iliac artery, *EVAR* endovascular aneurysm repair.

Out of a total of 482 patients with 964 iliac limbs, the main analysis included 141 iliac limbs (14.6%) from 91 patients, comprising 74 males and 17 females. Exclusion criteria are detailed in Fig. 2. None of the included patients had undergone primary EVAR for an infected (mycotic) AAA. Additionally, 8 patients (8.8%) in the final cohort had undergone primary EVAR for a ruptured infrarenal aortic aneurysm. The calculated statistical power for the final sample size of 141 iliac limbs in this study was 0.819, indicating the study’s ability to detect significant associations.

### Iliac limb migration

**Fig. 3:**
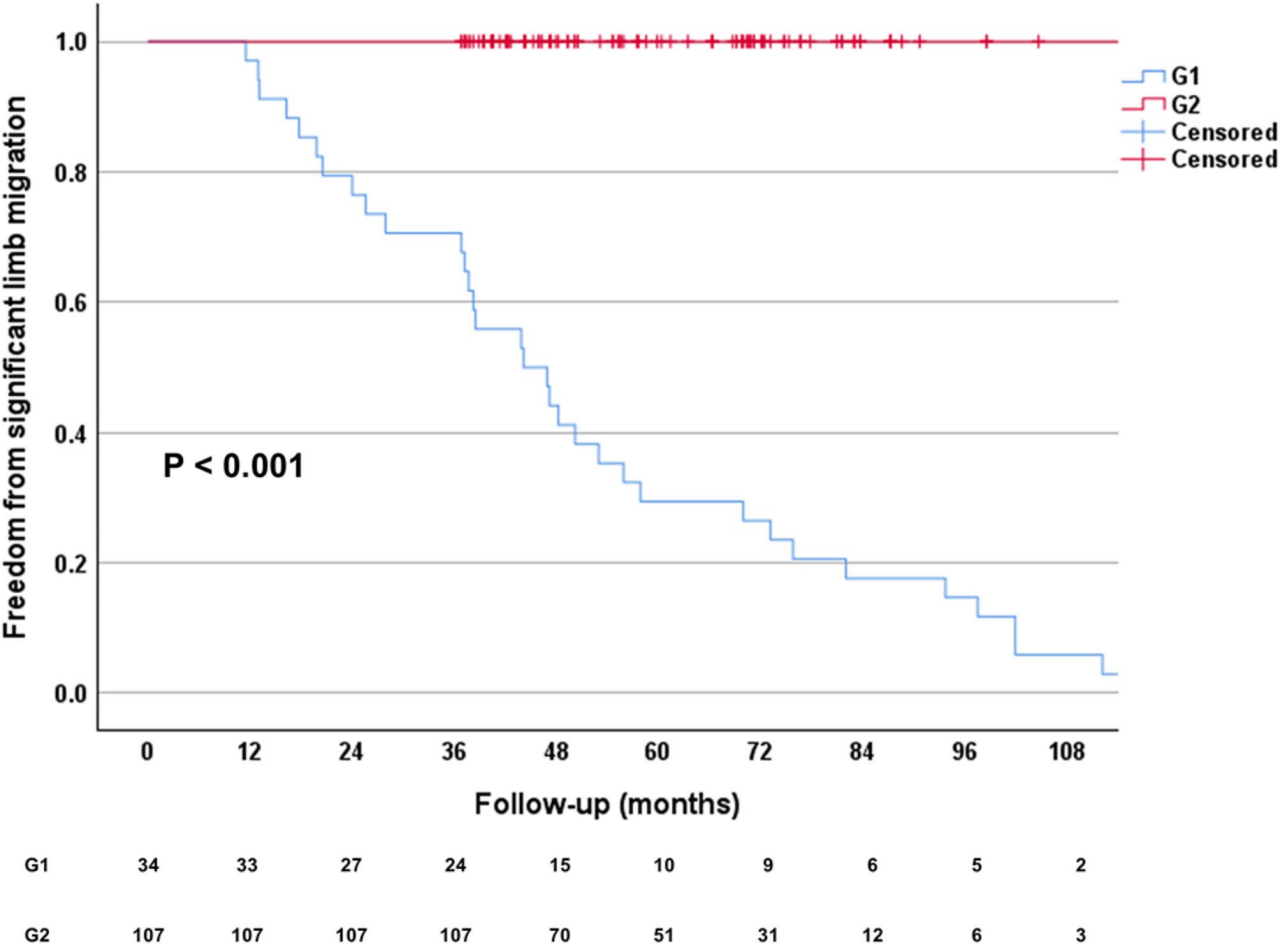
Kaplan-Meier survival analysis for the time to first detection of significant iliac limb migration following endovascular infrarenal aortic repair. The Kaplan-Meier curves illustrate the cumulative probability of remaining free from significant iliac limb migration (≥ 5 mm) over time. The table below the graph displays the number of iliac limbs at risk at each time interval. *G1*, group 1; *G2*, group 2.

Among the 141 iliac limbs examined, 34 (24.1%) were identified as having significant migration (≥ 5 mm) and were categorized as G1, while 107 limbs (75.9%) showed no significant migration (< 5 mm) and were classified as G2. All iliac limbs in G1 demonstrated upward migration. The median migration length for G2 was 2.0 mm (1.0–3.0 mm), while for G1, it was significantly greater at 8.0 mm (6.0–18.3 mm) (*P* < 0.001). The median follow-up period for the iliac limbs was 61.6 (45.9–76.2) months, with follow-up durations of 70.5 (49.7–91.8) months for G1 and 57.6 (44.2–73.2) months for G2 (*P* = 0.030). The median migration rate per year was 1.54 mm/year (1.01–2.63 mm/year) in G1 and 0.34 mm/year (0.15–0.55 mm/year) in G2 (*P* < 0.001). Kaplan-Meier survival analysis indicated that the median time to the first detection of significant iliac limb migration was 44.1 months (25.6–73.2 months) in G1 (*P* < 0.001) (Fig. 3). Additionally, other baseline demographic characteristics, including age, sex, limb site, smoking status, diabetes mellitus, hypertension, coronary artery disease, chronic kidney disease, and cerebrovascular accidents, showed no statistically significant differences between the two groups (Table 1).

**Table 1:** Comparative analysis of demographics, anatomical characteristics, and procedural factors between groups with (G1) and without (G2) significant limb migration. The data is displayed as median (25th–75th percentiles), number (%), or mean ± standard deviation. *AAA*, abdominal aortic aneurysm; *CIA*, common iliac artery; *EVAR* endovascular aneurysm repair.

Variables	G1 (*n* = 34)	G2 (*n* = 107)	*P* value
Median migration length (mm)	8.0 (6.0–18.3)	2.0 (1.0–3.0)	< 0.001
Median follow-up (months)	70.5 (49.7–91.8)	57.6 (44.2–73.2)	0.030
Median migration rate (mm/year)	1.54 (1.01–2.63)	0.34 (0.15–0.55)	< 0.001
Age (years)	76.0 (69.8–80.0)	77.0 (68.0–83.0)	0.447
Male sex	27 (79.4%)	87 (81.3%)	0.807
Ruptured infrarenal AAA before primary EVAR	1 (2.9%)	13 (12.1%)	0.188
Limb site (Left / Right)	20 (58.8%) / 14 (41.2%)	58 (54.2%) / 49 (45.8%)	0.637
Past smoking	3 (8.8%)	4 (3.7%)	0.359
Current smoking	6 (17.6%)	12 (11.2%)	0.328
Diabetes mellitus	6 (17.6%)	19 (17.8%)	0.988
Hypertension	20 (58.8%)	67 (62.6%)	0.692
Coronary artery disease	6 (17.6%)	25 (23.4%)	0.483
Chronic kidney disease	0 (0.0%)	7 (6.5%)	0.196
Cerebrovascular accident	8 (23.5%)	15 (14.0%)	0.191
**Anatomical characteristics**			
Infrarenal AAA diameter (cm)	6.0 (5.2–7.0)	5.5 (4.5–7.2)	0.289
Aortic tortuosity index	1.2 (1.1–1.3)	1.1 (1.1–1.3)	0.292
Infrarenal aortic angle (° )	122.9 (104.1–137.8)	134.0 (102.0–148.7)	0.131
CIA diameter (mm)	19.8 (16.1–23.7)	15.1 (13.7–18.5)	< 0.001
CIA length (mm)	49.5 (40.0–69.3)	47.0 (37.0–56.5)	0.243
Iliac tortuosity index	1.4 (1.3–1.5)	1.3 (1.3–1.5)	0.422
Iliac angle (° )	66.2 ± 33.4	83.8 ± 31.8	0.006
**Procedural factors**			
Iliac sealing length (mm)	39.80 ± 16.19	40.23 ± 18.84	0.904
Iliac sealing degree (%)	74.4 (64.8–89.3)	83.1 (70.5–94.4)	0.030
Iliac limb oversizing degree (%)	10.9 (7.6–18.8)	19.0 (13.0–26.7)	0.010
Iliac limb diameter (mm)	20.0 (16.0–20.0)	16.0 (14.5–20.0)	0.012
Ballerina configuration of iliac limbs	16 (47.1%)	54 (50.5%)	0.729
Iliac limb manufacturers			0.665
Medtronic Endurant (Santa Rosa, CA, USA)	14 (41.2%)	44 (41.1%)	
Gore Excluder (Flagstaff, AZ, USA)	15 (44.1%)	51 (47.7%)	
Cook Zenith (Bloomington, IN, USA)	3 (8.8%)	10 (9.3%)	
S&G Seal (Seongnam, Korea)	2 (5.9%)	2 (1.9%)	
Type 2 endoleak occurrence	16 (47.1%)	40 (37.4%)	0.315

### Anatomical and procedural factors

A comparative analysis of the anatomical characteristics and procedural factors of G1 and G2 is detailed in Table 1. The preoperative CIA diameter was significantly larger in G1 (median 19.8 mm, 16.1–23.7 mm) compared to G2 (median 15.1 mm, 13.7–18.5 mm) (*P* < 0.001). Additionally, the iliac angle was significantly more acute in G1 (66.2 ± 33.4°) compared to G2 (83.8 ± 31.8°) (*P* = 0.006). No significant differences were detected in other anatomical factors, including the preoperative diameter of the aortic aneurysm sac, aortic tortuosity index, infrarenal aortic angle, CIA length, and iliac tortuosity index. Among procedural factors, no statistically significant difference was observed in the length of the iliac seal between the two groups (*P* = 0.900). However, the degree of the iliac seal was significantly lower in G1 (median 74.4%, 64.8–89.3%) compared to G2 (median 83.1%, 70.5–94.4%) (*P* = 0.030). Moreover, the degree of iliac limb oversizing was significantly smaller in G1 (10.9%, 7.6–18.8%) compared to G2 (19.0%, 13.0–26.7%) (*P* = 0.010). In addition, the diameter of the iliac limb was significantly larger in G1 (median 20.0 mm, 16.0–20.0 mm) compared to G2 (median 16.0 mm, 14.5–20.0 mm) (*P* = 0.012). No significant difference was observed between the two groups in the occurrence of type 2 endoleaks, the manufacturers of the iliac limbs, or whether the iliac limbs were deployed in a Ballerina configuration.

### Risk factors for significant migration

Univariate analysis identified aortic tortuosity index, CIA diameter, iliac angle, iliac limb oversizing degree, and iliac limb diameter as significant predictors of iliac limb migration (Table 2). Pearson’s correlation analysis revealed a strong correlation between CIA diameter and iliac limb diameter (*r* = 0.711, *P* < 0.001), with a high VIF of 11.078. To address collinearity and ensure model stability, iliac limb diameter was excluded from the final multivariate Cox model. In the multivariable Cox proportional hazards model, CIA diameter (hazard ratio 1.16, 95% confidence interval 1.05–1.27, *P* = 0.002) and iliac limb oversizing degree (hazard ratio 0.97, 95% confidence interval 0.96–0.99, *P* = 0.002) remained significant predictors of significant iliac limb migration.

**Table 2:** Predictive factors for significant limb migration following endovascular infrarenal aortic repair. Univariate and multivariate Cox proportional hazards analyses were performed. Due to high collinearity between common iliac artery (CIA) diameter and iliac limb diameter (variance inflation factor = 11.078, Pearson’s *R* = 0.711, *P* < 0.001), only CIA diameter was included in the multivariate analysis. *AAA*, abdominal aortic aneurysm; *CI*, confidence interval; *CIA*, common iliac artery; *EVAR* endovascular aneurysm repair; *HR*, hazard ratio.

Variables	Univariable			Multivariable		
	HR	95% CI	*P* value	HR	95% CI	*P* value
Age	1.00	0.96–1.05	0.872			
Sex (female)	1.08	0.47–2.51	0.854			
Limb site (right)	1.02	0.51–2.04	0.948			
Ruptured infrarenal AAA before primary EVAR	0.38	0.05–2.79	0.340			
Past smoking	1.28	0.38–4.32	0.692			
Current smoking	1.48	0.61–3.59	0.392			
Diabetes mellitus	0.68	0.26–1.81	0.444			
Hypertension	0.93	0.47–1.85	0.835			
Coronary artery disease	0.90	0.36–2.22	0.816			
Chronic kidney disease	0.05	0.00–301.81	0.493			
Cerebrovascular accident	1.32	0.59–2.93	0.498			
**Anatomical characteristics**						
Infrarenal AAA diameter	1.06	0.88–1.27	0.561			
Aortic tortuosity index	11.31	1.06–120.96	0.045	4.10	0.28–59.37	0.301
Infrarenal aortic angle	0.99	0.98–1.00	0.093			
CIA diameter	1.12	1.03–1.21	< 0.001	1.16	1.05–1.27	0.002
CIA length	1.01	0.99–1.02	0.255			
Iliac tortuosity index	1.06	0.17–6.51	0.954			
Iliac angle	0.98	0.97–0.99	< 0.001	0.99	0.98–1.00	0.096
**Procedural factors**						
Iliac sealing length	1.01	0.99–1.02	0.547			
Iliac sealing degree	1.00	0.98–1.01	0.860			
Iliac limb oversizing degree	0.97	0.96–0.99	0.004	0.97	0.96–0.99	0.002
Iliac limb diameter	1.05	1.01–1.15	0.010			
Ballerina configuration of iliac limbs	1.17	0.59–2.30	0.646			
Iliac limb manufacturers						
Medtronic Endurant (Santa Rosa, CA, USA)	1.00					
Gore Excluder (Flagstaff, AZ, USA)	0.78	0.38–1.63	0.510			
Cook Zenith (Bloomington, IN, USA)	0.29	0.06–1.39	0.121			
S&G Seal (Seongnam, Korea)	0.80	0.17–3.78	0.780			
Type 2 endoleak occurrence	1.28	0.65–2.50	0.481			

**Fig. 4:**
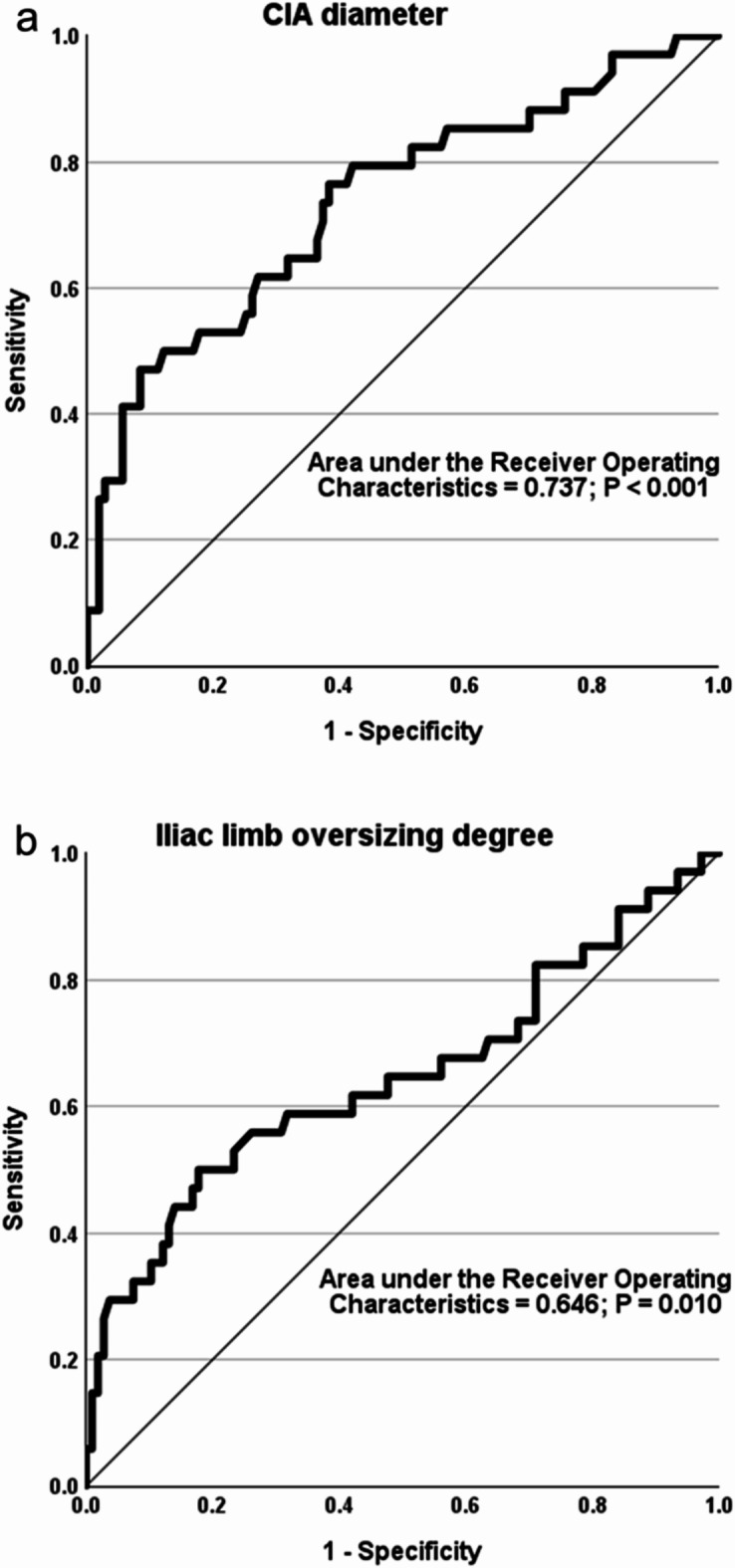
Receiver operating characteristic curves to identify the optimal cutoff values for (**a**) the diameter of the CIA and (**b**) the degree of oversizing of the iliac limb associated with an increased risk of significant limb migration. *CIA* common iliac artery.

**Fig. 5:**
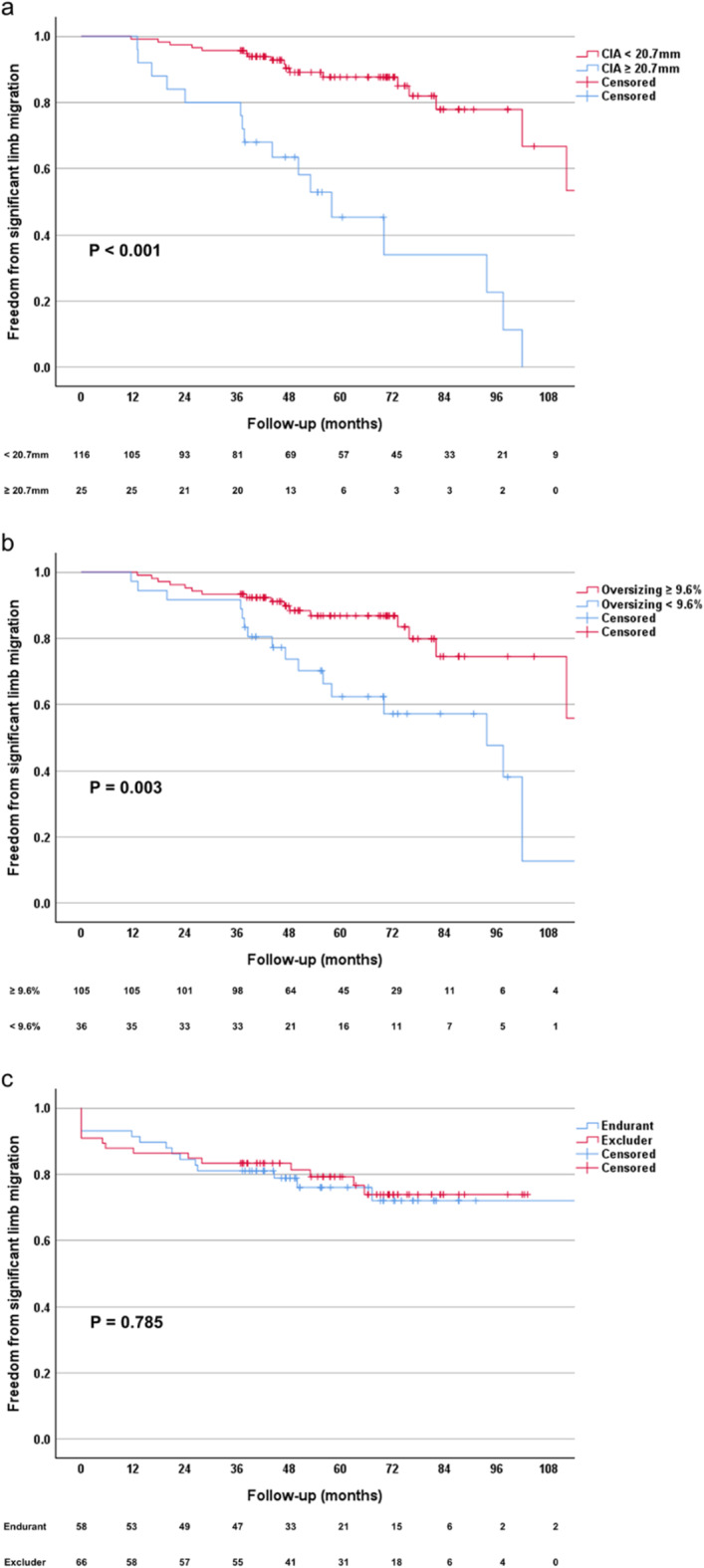
Kaplan-Meier survival analysis of significant iliac limb migration following endovascular infrarenal aortic repair. The Kaplan-Meier curves illustrate the likelihood of remaining free from significant limb migration based on (**a**) the preoperative diameter of the common iliac artery, (**b**) the degree of oversizing of the iliac limb, and (**c**) the type of stent-graft used (Medtronic Endurant vs. Gore Excluder). The tables below each graph indicate the number of iliac limbs without significant migration at designated time intervals post-procedure.

ROC curve analysis identified a CIA diameter of 20.7 mm (Youden’s index: 0.387) and iliac limb oversizing of 9.6% (Youden’s index: 0.322) as the optimal cutoff values associated with an increased risk of significant limb migration (Fig. 4). The CIA diameters and degrees of iliac limb oversizing were categorized into two groups based on these cutoff values. Subsequently, a Kaplan-Meier survival analysis was conducted. Iliac limbs with a preoperative CIA diameter of 20.7 mm or more, or iliac limb oversizing degree less than 9.6%, exhibited significantly higher rates of significant iliac limb migration during the follow-up period (Fig. 5a and b). A subgroup analysis comparing the two most frequently used stent-graft types revealed significant migration in 14 out of 58 iliac limbs (24.1%) for the Medtronic Endurant (Santa Rosa, CA, USA) and in 15 out of 66 iliac limbs (22.7%) for the Gore Excluder (Flagstaff, AZ, USA). The mean time to significant migration was 96.8 months (95% confidence interval 84.4–109.2) for the Medtronic Endurant and 82.9 months (95% confidence interval 73.7–92.0) for the Gore Excluder. Kaplan-Meier analysis indicated no statistically significant difference in the freedom from significant iliac limb migration between the two stent-graft types (*P* = 0.785), as illustrated in Fig. 5c.

### Endoleak occurrence

**Fig. 6:**
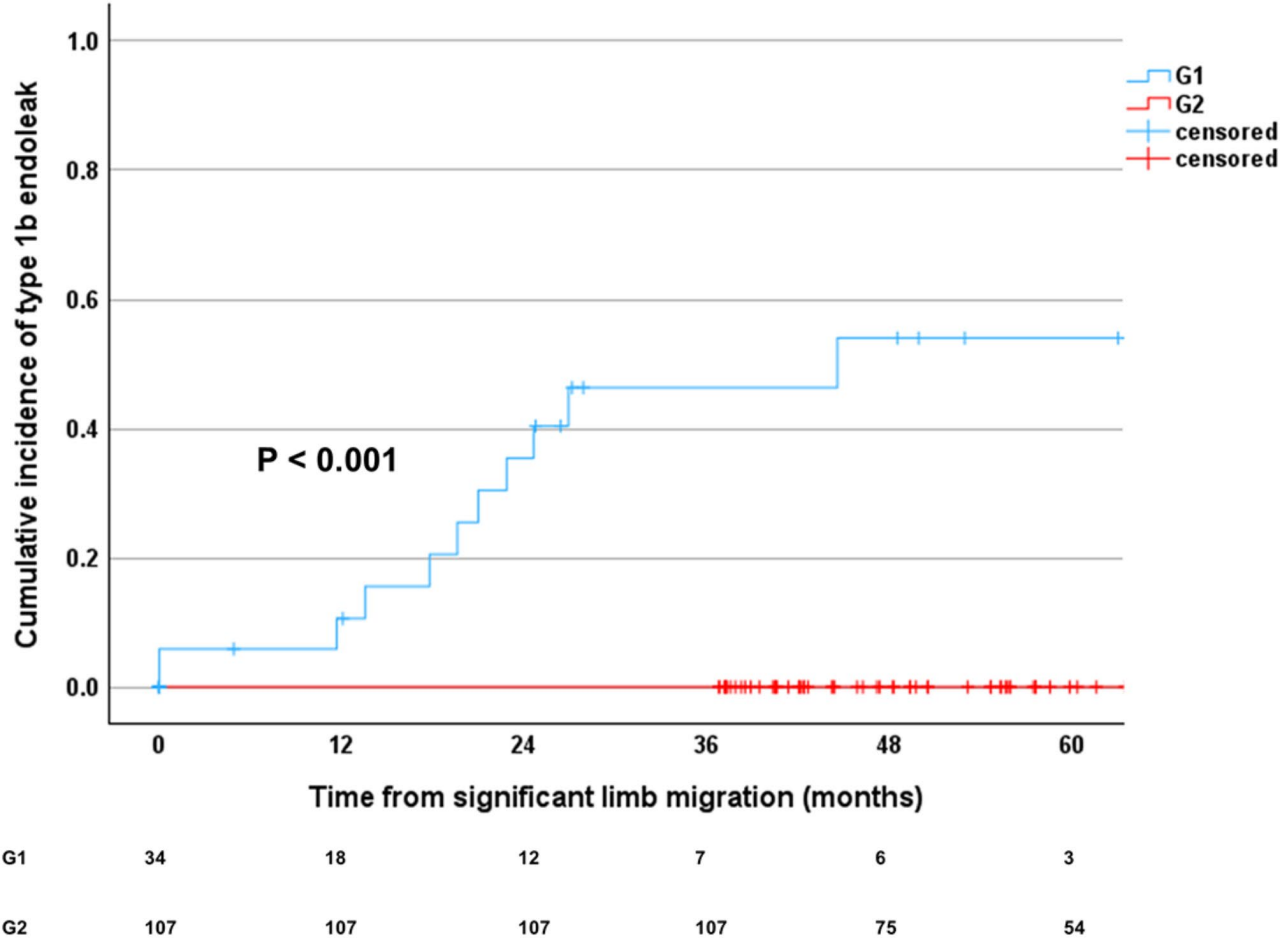
Kaplan-Meier survival analysis of cumulative incidence of type 1b endoleaks following significant iliac limb migration. The Kaplan-Meier curves illustrate the cumulative incidence of type 1b endoleaks after significant iliac limb migration for G1 and G2. The table below the graph indicates the number of iliac limbs at risk at specific time intervals post-migration. *G1*, group 1; *G2*, group 2.

Type 1b endoleaks were not observed in G2. Among the iliac limbs in G1, 11 out of 34 limbs (32.4%) had type 1b endoleaks (odds ratio 5.652, 95% confidence interval 3.901–8.189, *P* < 0.001). Type 1b endoleaks were first detected on average 59.9 ± 24.0 months post-EVAR. Among iliac limbs exhibiting significant migration (G1), the median time from the onset of significant migration to the detection of type 1b endoleaks was 15.7 months (0–27.3 months) (Fig. 6). A statistically significant difference was observed between G1 and G2 (*P* < 0.001). In iliac limbs that developed type 1b endoleaks, the median time to significant iliac limb migration was 44.1 months (16.3–53.0 months), whereas the median time to the detection of type 1b endoleaks was 59.9 months (37.8–73.5 months). A strong positive correlation (*r* = 0.927, *P* < 0.001) was identified between the time to significant migration and the time to type 1b endoleak detection, indicating a close temporal association between these events. Of the 11 limbs with type 1b endoleaks, 10 were identified as having type 1b endoleaks more than three years post-EVAR and subsequently underwent iliac limb extension as a reintervention. The remaining iliac limb showed a type 1b endoleak at 31.3 months post-EVAR. Despite multiple recommendations for reintervention based on clinical guidelines, the patient strongly refused further treatment and opted for continued surveillance. This decision was made after the patient was fully informed of the potential risks, including aneurysm sac expansion and rupture. Type 1a or 3 endoleaks were not observed in either group.

### Changes in diameter of CIA and its relationship with iliac limb migration and Type 1b endoleak

A comparative analysis of changes in CIA diameter between the study groups is presented in Table 3. The last follow-up CIA diameter was significantly larger in G1 (median 27.7 mm, 21.6–33.8 mm) compared to G2 (median 17.8 mm, 15.7–21.3 mm) (*P* < 0.001). Furthermore, the change in CIA diameter was greater in G1 (median 5.9 mm, 3.2–12.1 mm) than in G2 (median 2.4 mm, 1.3–3.4 mm) (*P* < 0.001).

**Table 3:** Comparison of common iliac artery (CIA) diameter at baseline and follow-up between groups with and without significant iliac limb migration (G1 vs. G2) and with and without type 1b endoleak (G3 vs. G4). The change in CIA diameter was calculated as follows: last follow-up CIA diameter minus pre-procedural CIA diameter. The data is displayed as median (25th–75th percentiles). *CIA*, common iliac artery.

Variables	G1 (*n* = 34)	G2 (*n* = 107)	*P* value	G3 (*n* = 11)	G4 (*n* = 130)	*P* value
Pre-procedural CIA diameter (mm)	19.8 (16.1–23.7)	15.1 (13.7–18.5)	< 0.001	18.3 (16.9–21.4)	15.5 (13.8–19.5)	0.106
Last follow-up CIA diameter (mm)	27.7 (21.6–33.8)	17.8 (15.7–21.3)	< 0.001	30.8 (22.9–36.2)	18.7 (16.1–22.6)	< 0.001
Change in CIA diameter (mm)	5.9 (3.2–12.1)	2.4 (1.3–3.4)	< 0.001	12.5 (3.2–15.0)	2.6 (1.4–4.2)	< 0.001

Among iliac limbs that developed a type 1b endoleak (G3), the last follow-up CIA diameter was significantly larger (median 30.8 mm, 22.9–36.2 mm) compared to those without a type 1b endoleak (G4) (median 18.7 mm, 16.1–22.6 mm) (*P* < 0.001). The change in CIA diameter was also significantly greater in G3 (median 12.5 mm, 3.2–15.0 mm) than in G4 (median 2.6 mm, 1.4–4.2 mm) (*P* < 0.001). However, there was no significant difference in preoperative CIA diameter between G3 and G4 (*P* = 0.106).

Spearman’s correlation analysis revealed a moderate positive correlation between the length of iliac limb migration and changes in CIA diameter (*r* = 0.344, *P* < 0.001). Similarly, a positive correlation was observed between changes in CIA diameter and the occurrence of type 1b endoleak, as assessed using both Kendall’s tau-b (τ = 0.269, *P* < 0.001) and Spearman’s rho (*r* = 0.327, *P* < 0.001).

## Discussion

Our study demonstrated that a large CIA diameter and a low degree of iliac limb oversizing are significant risk factors for late migration of the iliac limb. Additionally, we found a significantly higher incidence of type 1b endoleak in cases with significant iliac limb migration. The group with significant iliac limb migration also exhibited larger CIA diameters, more acute iliac angles, shorter iliac sealing degrees, and lower degrees of iliac limb oversizing compared to the group without significant migration. Furthermore, iliac limbs that experienced significant migration had larger pre-procedural and last follow-up CIA diameters compared to those without migration. Similarly, iliac limbs that developed type 1b endoleaks exhibited greater changes in CIA diameter over time, despite no significant difference in preoperative CIA diameter between those with and without type 1b endoleaks. Correlation analysis further revealed a positive association between changes in CIA diameter and both the length of iliac limb migration and the occurrence of type 1b endoleaks.

Previous studies have primarily focused on identifying risk factors associated with the need for reinterventions or the occurrence of type 1b endoleaks, with limited analysis of iliac limb migration at the distal landing zone^8,20,21^. Our research focused on examining the risk factors contributing to significant migration of the iliac limb at the distal landing zone. This specific emphasis is clinically significant because iliac limb migration at the distal landing zone is a key factor necessitating reinterventions after EVAR^8^.

To the best of our knowledge, limited research directly compares to our findings on the risk factors influencing iliac limb migration. One study suggested that factors such as large AAA diameters, dilated or aneurysmal CIAs, short lengths of iliac fixation, lower degrees of iliac limb oversizing, and bell-bottom iliac limbs could influence iliac limb migration^13^. However, this study was limited by its small sample size of only four patients, which precluded meaningful statistical analysis. Iliac stent graft displacement forces were analyzed in an experimental model, showing significant forces at the distal end of bell-bottom grafts; however, the study was conducted in a controlled laboratory setting^14^. Mallios et al. reported a case of proximal iliac limb migration post-EVAR, successfully treated with a branched iliac device, but did not analyze the contributing factors^15^. Bastos Gonçalves et al. investigated the dynamics of iliac attachment zones following EVAR, reporting iliac limb retraction (≥ 5 mm) in 9.1% of cases^16^. They identified the implantation of iliac limb stent grafts measuring ≥ 24 mm and iliac artery dilation exceeding the graft diameter as independent risk factors for retraction. Furthermore, their findings established a temporal relationship between progressive iliac dilatation, iliac limb retraction, and clinically significant seal loss, reinforcing the role of iliac dilatation and limb retraction in seal zone-related complications.

Prior research has shown a correlation between the diameter of the CIA and the degree of iliac limb oversizing with the occurrence of type 1b endoleaks. Gibello et al. identified a statistically significant increase in the occurrence of late type 1b endoleak among individuals with a CIA diameter equal to or larger than 18 mm within a study group of 648 patients^22^. More precisely, the prevalence of late type 1b endoleak in patients with an enlarged CIA was 7.2% at a mean follow-up period of 74.8 months, compared to 3.2% in patients with a normal CIA (*P* = 0.01). Similarly, Nana et al. demonstrated a significantly higher incidence of type 1b endoleak at 12 months in individuals with a CIA diameter of 18 mm or more, with a prevalence of 5.3%, compared to 0.5% in those with a CIA diameter less than 18 mm (*P* = 0.04)^23^. In another study, Mascoli et al. reported that an iliac limb oversizing of less than 10% was linked to a significant increase in the risk of late type 1b endoleak (odds ratio 5.4, 95% confidence interval 1.3–21.5, *P* = 0.01)^24^. Furthermore, research investigating predisposing factors for reintervention with additional iliac stent grafts after EVAR has shown that limbs requiring reintervention had larger CIA diameters (median 18 mm, 25th and 75th percentile 20–25 mm) than those that did not require reintervention (median 15 mm, 13–18 mm, *P* < 0.001)^8^. Additionally, this study found a lower degree of oversizing of the iliac limb in re-intervened patients during the initial procedure (median 11% versus 18%, *P* = 0.003). Significant migration of the iliac limb was identified as a major factor contributing to the need for reintervention in the distal landing zone^8^. The findings of Bastos Gonçalves et al. further reinforce these observations, demonstrating that iliac dilatation and retraction frequently occur after EVAR and are significantly associated with seal zone complications^16^. Their study underscores the importance of optimizing iliac limb oversizing and ensuring adequate distal seal length to mitigate the risks of migration and long-term seal failure. Our results, combined with these prior studies, indicate that aneurysmal CIA and inadequate oversizing of the iliac limb are correlated with significant iliac limb migration. This migration, along with progressive iliac dilatation, may subsequently increase the risk of late type 1b endoleaks and necessitate additional interventions.

Other established variables that may impact the development of type 1b endoleak include the initial diameter of AAA, the length of the CIA landing zone, and iliac tortuosity^25^. In their study of 259 CIAs as distal landing zones, Choi et al. noted a significantly higher incidence of late type 1b endoleak in patients with larger maximum AAA diameters, shorter CIAs, and shorter distal sealing lengths during the follow-up period (*P* < 0.001, *P* = 0.02, *P* = 0.002, respectively)^21^. Moreover, Kaladji et al. found that a high preoperative iliac tortuosity index is significantly associated with the occurrence of type 1b endoleak (*P* = 0.011)^26^. In our research, we found that the initial diameter of AAA, the length of the CIA landing zone, and the iliac tortuosity index did not show a statistically significant relationship with significant migration of the iliac limb. This discrepancy between our findings and previous studies is presumed to arise from the different focuses of the research. While our study concentrated on factors influencing significant iliac limb migration, previous studies examined factors affecting the occurrence of type 1b endoleaks. The exact cause of delayed type 1b endoleak is not fully understood. However, it is believed that, in addition to significant migration of the iliac limb at the distal landing zone, factors such as dilatation of the iliac artery landing site and insufficient distal attachment lengths of stent-grafts within the CIA may contribute to the susceptibility for late type 1b endoleak development^25^. Various factors, such as the initial diameter of the AAA, the length of the landing zone of the CIA, and the iliac tortuosity index are hypothesized to influence mechanisms other than significant migration of the iliac limb in the development of late type 1b endoleak.

In our study, the absolute iliac sealing length did not differ significantly between groups; however, the sealing degree (%) was significantly lower in limbs with significant migration. Anatomical variations in the distal sealing zone, such as vessel tapering, tortuosity, and calcification, may contribute to differences in the sealing degree despite similar absolute lengths. Previous studies have emphasized the importance of achieving a distal sealing length of at least 20 mm to reduce the risk of late type 1b endoleaks^21^. However, the present study included patients with considerably longer sealing lengths (mean: 39.80 ± 16.19 mm in G1, 40.23 ± 18.84 mm in G2), highlighting the importance of securing an adequate absolute sealing length in endograft stability. Nevertheless, the significant difference in the sealing degree between groups underscores the additional importance of achieving optimal circumferential sealing coverage, even when the absolute length is sufficient. Clinically, these findings suggest that while securing a minimum absolute sealing length is essential, optimizing the sealing degree may be equally important in procedural planning to minimize the risk of migration and the development of late type 1b endoleaks. Further studies are warranted to clarify the interplay between these factors and their long-term impact on clinical outcomes.

Given that current post-EVAR monitoring protocols predominantly focus on AAA sac size changes and detecting endoleaks^1^, our study, which specifically investigates the iliac artery landing zone, could have significant clinical implications. Roos et al. reported that most patients who underwent reintervention with additional iliac stent grafts did not show an increase in AAA diameter^8^. Similarly, Schlösser et al. found that most delayed ruptures following EVAR occurred without aneurysm expansion on subsequent CT scans^27^. These findings, combined with our results, suggest that EVAR surveillance should consider not only changes in AAA sac diameter but also the condition of the iliac landing zone. Our research indicates that using a flared limb in EVAR for patients with a CIA diameter exceeding 20 mm is linked to a higher probability of significant iliac limb migration. Hence, comprehensive and vigilant monitoring of the iliac landing zone is crucial in such cases. Moreover, during the procedure, it is essential to intentionally oversize the iliac limbs adequately when placing them into a wider CIA. However, excessive oversizing should be avoided, as a study has demonstrated that exceeding a 15% oversizing threshold increases the likelihood of limb occlusion^28^.

The primary limitation of this study is its retrospective design, which resulted in the exclusion of a significant portion of the initial cohort. Due to the absence of follow-up CTA data beyond three years post-EVAR, only less than 15% of the initial population was included in the final analysis. This high exclusion rate may have influenced the reported occurrence of type 1 endoleaks. Since only patients with a minimum of three years of CTA follow-up data were included, patients who experienced early type 1 endoleaks may have been lost to follow-up due to aneurysm rupture or other complications before reaching the three-year mark. Despite the final sample of 141 iliac limbs meeting statistical power requirements, the high exclusion rate of 85% may limit the generalizability of the findings. Excluded participants may differ systematically from those included, introducing selection bias. Moreover, the extensive inclusion period (spanning over a decade) resulted in variability in follow-up durations among patients, which could potentially introduce bias into the analysis of iliac limb migration. Although the Cox proportional hazards model inherently accounts for time-to-event differences, residual bias related to follow-up duration may still affect the assessment of significant iliac limb migration. Additionally, the retrospective design led to variability in follow-up CTA intervals. Although all iliac limbs were assessed for migration using the initial post-operative CTA and the last follow-up CTA, only those exhibiting significant migration (≥ 5 mm) underwent additional interim CTA assessments. Consequently, CTA scans obtained between the initial post-operative assessment and the last follow-up in cases without significant migration were not analyzed. The follow-up intervals for migration measurements varied among patients, potentially affecting the timing of event detection. This inconsistency may have introduced variability in migration reporting, impacting the accuracy of comparisons between groups. However, our approach facilitated a systematic evaluation of significant iliac limb migration while remaining feasible within a retrospective study design. Additionally, this study used the origin of the deep femoral artery as the reference point, rather than the origin of the hypogastric artery, for measuring the iliac limb migration distance. This decision was made because, particularly in the initial post-operative CTA scans conducted one week after the procedure, the orifice of the hypogastric artery was often too close to the distal end of the iliac limb stent-graft. This proximity complicated the acquisition of accurate measurements using the central lumen-line reconstruction methodology on a dedicated workstation. Although employing the deep femoral artery as the reference point facilitated consistent measurements, it resulted in a longer measurement distance to the distal end of the iliac limb stent-graft compared to using the hypogastric artery. Consequently, this approach may have increased the potential for measurement error, which should be considered when interpreting the study findings. Finally, our study did not include an analysis of CIA calcification or thrombosis, both of which may influence the stability of the distal seal zone and the risk of iliac limb migration. A previous study suggested that CIA calcification is associated with early type 1b endoleak but not with late type 1b endoleak, while CIA thrombosis does not appear to have a significant correlation with either early or late type 1b endoleak^21^. However, the role of these factors in significant iliac limb migration remains uncertain. Future studies incorporating an evaluation of distal landing zone calcification and thrombosis could provide further insights into their potential impact on iliac limb stability and late complications following EVAR.

In conclusion, our study established that the diameter of the CIA and the degree of iliac limb oversizing are significantly correlated with iliac limb migration. Moreover, significant migration of the iliac limb significantly increases the risk of developing a type 1b endoleak. Based on these findings, we recommend operators ensure careful oversizing of the iliac limbs by at least 10% during procedures for patients with a CIA diameter greater than 20 mm. Furthermore, comprehensive and vigilant monitoring of the iliac landing zone is crucial during postoperative surveillance for these patients who have undergone EVAR with a flared limb.

## Data Availability

The datasets used and/or analysed during the current study available from the corresponding author on reasonable request.
